# Targeting ERβ to fight melanoma: a new valid approach?

**DOI:** 10.1186/s12967-022-03358-y

**Published:** 2022-04-05

**Authors:** Marzia Di Donato, Antimo Migliaccio, Gabriella Castoria

**Affiliations:** Department of Precision Medicine, University of Campania ‘L. Vanvitelli’, Via L. De Crecchio, 7, 80138 Naples, Italy

Dear Editor,

The impact of sex steroids and their cognate receptors in many human cancers has been almost neglected for many years.

Gender disparities in melanoma, with a female advantage in its incidence and outcome have been reported [1]. However, the molecular aspects of these findings remain still pending, with few reports so far collected on the role of estrogen receptors (ERs), alpha (ERα) or beta (ERβ), or G-protein coupled estrogen receptor (GPER) in this cancer. ERs both mediate estrogen signaling through genomic or non-genomic mechanism, which might cooperate each other to regulate a range of responses in target tissues and human cancers [2]. The subsequent discovery that an orphan GPCR (GPR30, then renamed GPER) is required for rapid estrogen signaling opened new perspectives in estrogen biology and hormone-dependent cancers [3]. ERβ can be detected in benign nevi, pre-malignant and malignant melanocytic lesions. As such, it might represent a hallmark of melanoma progression. A role for GPER has been also proposed in differentiation and growth inhibition of melanoma cells, as well as their susceptibility to immune clearance [3]. GPER increased expression is correlated with reduced overall survival (OS) in melanoma patients [4]. Figure 1 resumes this scenario.

**Fig. 1:**
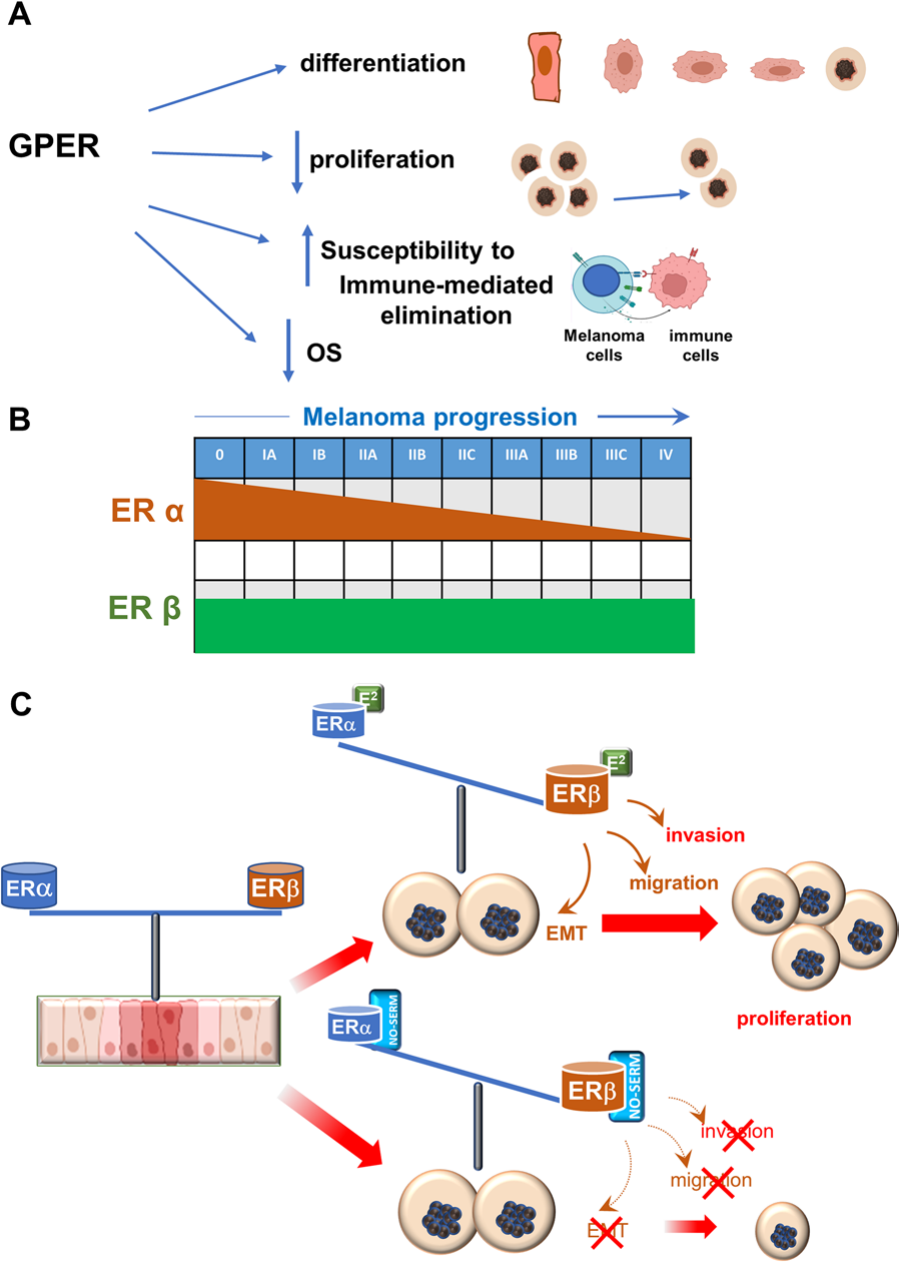
Schematic representation of GPER and ERs functions in melanoma. Targeting ERβ with a new SERM. **A** GPER promotes the differentiation and inhibits the proliferation of melanoma cells. It contributes to the susceptibility to immune-mediated killing and its expression is inversely related to overall survival (OS). **B** ERα and ERβ genes expression during the different stages of melanoma progression. **C** ERα and ERβ are both expressed during early stages of melanoma (left side). The unbalanced expression of ERβ correlates with invasion, migration and Epithelial-Mesenchymal Transition (EMT) induced by Estradiol (E2) in melanoma cells, which can be inhibited by a new SERM, with a NO-releasing moiety (NO-SERM) and higher selectivity for ERβ

Nevertheless, the role of ERs and GPER in melanoma is poorly investigated.

The paper recently published by Bechmann et al. [4] reports that ERβ gene expression does not change with melanoma progression, while sustained ERα gene expression correlates with extension of OS in a retrospective study from 448 patients. ERβ might be, hence, clinically actionable in melanoma. To this end, the Authors propose the use of NO-SERM 4d, a selective ERβ modulator (SERM) with a nitric oxide (NO)-releasing moiety. The compound combines the increased selectivity towards ERβ with the release of NO, which would mitigate the cardiovascular side effects exerted by SERMs [4]. NO-SERM 4d reduces the pro-metastatic behavior of melanoma-derived cells and impairs their growth in 3D models. In addition to supporting a oncogenic role of ERβ in melanoma, the paper reinforces the concept that changes in ER (α or β) levels may limit or expand the proliferative and metastatic potential of human cancers. Remarkably, the selective targeting of ERβ might be used as a novel, useful approach in melanoma therapy.

The report, however, leaves still open several questions. The first concerns the molecular mechanism linking ERβ with melanoma growth and spreading. Is it genomic or non-genomic? Or even, does it require the integration of the two aforementioned mechanisms with GPER-mediated responses? The second, no less important question, regards the identification of ERβ isoform(s) involved in melanoma progression, as in humans there are at least five ERβ isoforms (ERβ 1, 2, 3, 4, 5), and among them only ERβ1 is functional, while the others control its activity [5]. Again, despite the female advantage in melanoma incidence and progression, it still remains unclear how and when estrogens achieve a significant concentration to activate the α or β ER isoforms in melanoma cells. This issue is strengthened by the possibility that a local increase of 3β-androstanediol, the natural ERβ ligand, specifically activates ERβ to foster melanoma’s malignancy. Investigation of 5α-reductase enzymes that convert the dihydrotestosterone into 3β-androstanediol, might be hence envisaged, as the 5α-reductase type 1 can be found in skin and melanoma cells. At last, fluctuations in serum levels and ratio of sex steroids might significantly affect the clinical course of melanoma, as it occurs in prostate cancer. All these questions remain unanswered, together with in depth understanding of the role of other sex-steroid receptors in melanoma onset and pathogenesis.

Despite these arising matters, the clinical relevance of reported findings is undeniable. Results with NO-SERM 4d would at least integrate two ongoing clinical trials. The first one (NCT00492505) utilizes the combinatorial use of the ER antagonist, tamoxifen with sorafenib and cisplatin in stage III melanoma patients, while the second one (NCT00489944) provides the combination of tamoxifen with sunitinib and cisplatin in patients with high-risk ocular melanoma. Thus, the data reported by Becham et al. would significantly expand the repertoire of drugs used in the therapy of advanced melanoma and/or its susceptibility to chemo- or radiotherapy. The reported findings might also provide significant hints of whether SERMs engage their targets or eventually induce side-effects and/or resistance.

## Data Availability

Not applicable.
